# Effect of Sipjeondaebo-Tang on Cancer-Induced Anorexia and Cachexia in CT-26 Tumor-Bearing Mice

**DOI:** 10.1155/2014/736563

**Published:** 2014-05-14

**Authors:** Youn Kyung Choi, Ki Yong Jung, Sang-Mi Woo, Yee Jin Yun, Chan-Yong Jun, Jong Hyeong Park, Yong Cheol Shin, Sung-Gook Cho, Seong-Gyu Ko

**Affiliations:** ^1^Department of Preventive Medicine, College of Korean Medicine, Kyung Hee University, 1 Hoegi-dong, Dongdaemun-gu, Seoul 130-701, Republic of Korea; ^2^Department of Oriental Internal Medicine, College of Oriental Medicine, Gachon University, Seongnam, Republic of Korea

## Abstract

Cancer-associated anorexia and cachexia are a multifactorial condition described by a loss of body weight and muscle with anorexia, asthenia, and anemia. Moreover, they correlate with a high mortality rate, poor response to chemotherapy, poor performance status, and poor quality of life. Cancer cachexia is regulated by proinflammatory cytokines such as interleukin-6 (IL-6), monocyte chemoattractant protein-1 (MCP-1), and tumor necrosis factor-**α** (TNF-**α**). In addition, glucagon like peptide-1 (GIP-1), peptide YY (PYY), ghrelin, and leptin plays a crucial role in food intake. In this study, we investigated the therapeutic effects of one of the traditional herbal medicines, Sipjeondaebo-tang (Juzen-taiho-to in Japanese; SJDBT), on cancer anorexia and cachexia in a fundamental mouse cancer anorexia/cachexia model, CT-26 tumor-bearing mice. SJDBT was more significantly effective in a treatment model where it was treated after anorexia and cachexia than in a prevention model where it was treated before anorexia and cachexia on the basis of parameters such as weights of muscles and whole body and food intakes. Moreover, SJDBT inhibited a production of IL-6, MCP-1, PYY, and GLP-1 and ameliorated cancer-induced anemia. Therefore, our *in vivo* studies provide evidence on the role of SJDBT in cancer-associated anorexia and cachexia, thereby suggesting that SJDBT may be useful for treating cancer-associated anorexia and cachexia.

## 1. Introduction

Cancer anorexia and cachexia correlate with a high mortality rate, poor response to chemotherapy, poor performance status, and poor quality of life (QOL), particularly in physical, psychological, and social functions in cancer patients [1, 2]. Anorexia, which is characterized by the loss of desire to eat or loss of appetite, causes weight loss and malnutrition in cancer patients [3, 4]. Cachexia is a multifactorial condition described by muscle loss, which is in part associated with loss of body weight, anorexia, asthenia, and anemia [5, 6]. Cachexia occurs in about half of all cancer patients, and lung and upper gastrointestinal tract cancer patients have the highest frequency of cachexia, whereas patients with breast and lower gastrointestinal tract cancer have the lowest frequency of cachexia [7]. In addition, cachexia affects an ability of cancer patients to sustain chemotherapy or radiotherapy [8]. Therefore, it is important to manage anorexia and cachexia in cancer patients. However, current therapies for cancer patients with anorexia and cachexia are limited due to both poor efficacies and side effects of chemotherapeutics [9]. So, different therapeutic options are required to prevent cancer anorexia and cachexia.

Sipjeondaebo-tang (Juzen-taiho-to in Japanese and Shi-Quan-Da-Bu-Tang in Chinese; SJDBT), commonly used traditional herbal medicines in Korea, China, and Japan [10], is composed of 10 species of herbs. SJDBT is prescribed for patients suffering from anemia, fatigue, anorexia, scaly skin, and dryness of the mouth [11–13]. In addition, SJDBT has been known to have anticancer effects [14–19]. Nevertheless, its effect on cancer cachexia is poorly understood. Here, we examined the effect of SJDBT on key parameters of cancer anorexia and cachexia and found that SJDBT ameliorated cancer anorexia and cachexia* in vivo *by altering body and muscle weights, food intakes, and levels of cytokines, hormones, RBC, Hb, and HCT in CT-26 tumor-bearing mice suffering from anorexia and cachexia, thereby suggesting that SJDBT may be useful for treating cancer anorexia and cachexia.

## 2. Materials and Methods

### 2.1. Preparation of SJDBT

SJDBT were prepared by and obtained from Hanpoong Pharmaceutical Company (Jeonju, Korea) following the good manufacturing practices (GMP) procedures. SJDBT is manufactured as dried powder of hot water extract obtained from 10 herbs (320 g of* Angelica gigas*, 320 g of* Astragalus membranaceus*, 320 g of* Atractylodes japonica*, 320 g of* Cinnamomum cassia*, 320 g of* Cnidium officinale*, 160 g of* Glycyrrhiza uralensis*, 320 g of* Paeonia lactiflora*, 320 g of* Panax ginseng*, 320 g of* Poria cocos,* and 320 g of* Rehmannia glutinosa*; average yield = 28.85%). The dried powders were lyophilized and then dissolved the three different doses (low dose: 6.784 mg/kg, middle dose: 67.84 mg/kg, and high dose: 678.4 mg/kg) in distilled water.

### 2.2. Animal Study for Cancer Cachexia

**Figure 1:**
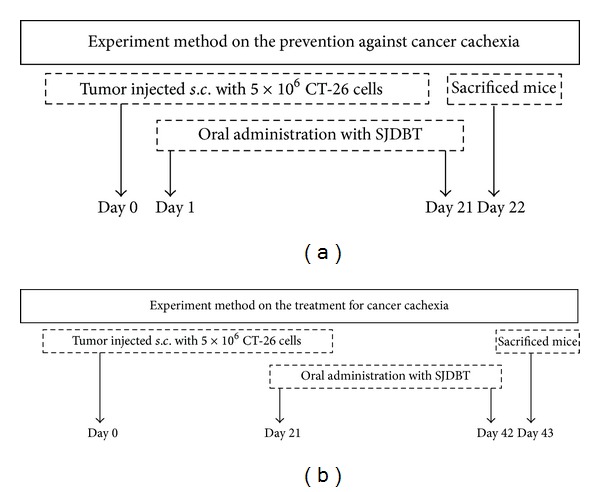
Schedules for prevention and therapy models of mouse cancer-induced cachexia. 5 × 10^6^ CT-26 tumor cells were subcutaneously (*s.c.*) injected into the right flank of BALB/c mice. (a) Experiment method on the prevention: a day after tumor cell inoculation, three doses of SJDBT (L-SJDBT of 6.784 mg/kg, M-SJDBT of 67.84 mg/kg, and H-SJDBT of 678.4 mg/kg) were* p.o.* administrated daily for 21 days. At 22 days after tumor cell injection, mice were sacrificed. (b) Experiment method on the therapy: 21 days after tumor cell inoculation (a day when cachexia was held), three different doses of SJDBT (L-SJDBT of 6.784 mg/kg, M-SJDBT of 67.84 mg/kg, and H-SJDBT of 678.4 mg/kg) were* p.o.* administrated daily for 21 days. At the end of experiments (42 days after tumor cell injection), mice were sacrificed.

Male BALB/c mice were purchased from Central Lab Animal Inc. (Seoul, Korea) at 6 weeks of age, and all mice were kept in pathogen-free environment. Mice were randomized into two groups (prevention or therapy model of cachexia). Each group was divided into subgroups (5 prevention groups: normal, control, low dose SJDBT (L-SJDBT of 6.784 mg/kg), middle dose SJDBT (M-SJDBT of 67.84 mg/kg), and high dose SJDBT (H-SJDBT of 678.4 mg/kg); 6 treatment groups: normal, control, L-SJDBT, M-SJDBT, H-SJDBT, and megestrol acetate- (MA-) treated). Animal studies were approved by the animal care center of Kyung-Hee University (KHUASP (SE)-12-048). The murine CT-26 tumor-bearing mouse was widely used for cancer cachexia model [20]. CT-26 colon carcinoma cell was obtained from the American Type Culture Collection (ATCC) (Manassas, VA, USA) and from Dr. Suk-Chan Lee (Department of Genetic Engineering, Sungkyunkwan University, Suwon, Korea). Cells were cultured in RPMI-1640 medium with 10% fetal bovine serum and 1% antibiotics. The schematic experimental procedure is described in Figure 1. For the prevention model of cancer-induced anorexia and cachexia, mice were injected* s.c.* with CT-26 cells (5 × 10^6^). A day after tumor cell injection, three different doses of SJDBT (L-SJDBT, M-SJDBT, or H-SJDBT) were* p.o.* added daily for 21 days. For the treatment model of cancer-induced anorexia and cachexia, mice were injected* s.c.* with CT-26 cells (5 × 10^6^) and then three different doses of SJDBT (L-SJDBT, M-SJDBT, or H-SJDBT) were* p.o.* added daily for 21 days at 3 weeks after tumor cell injection (Figure 6). MA (Santa Cruz Biotechnology, CA, USA) was used as a positive control for anorexic effect and dissolved in corn oil (100 mg/kg).

### 2.3. Measurement of Food Intakes and Weights of Whole Body and Muscles

Body weight and food intake were measured every day using an electronic scale. The measured quantity of food intake was divided by the number of mice to determine each intake per animal per day. At the time of sacrifice, the gastrocnemius muscles were dissected and weighted.

### 2.4. Measurement of Levels of Cytokines and Hormones and Blood Analysis

Whole blood samples were collected by cardiac puncture, and serum was obtained after being centrifuged from the whole blood. Cytokines and hormones level were measured using a Milliplex Mouse Metabolic Magnetic Bead Panel MMHMAG-44K-14 (Millipore, MO, USA) in a Luminex 200. Standards were plotted and concentrations were determined using Milliplex Analyst software version 5.1. The blood samples were placed in Vacutainer TM tubes containing EDTA (BD Science, NJ, USA). Blood analysis was performed using the HEMAVET 950 hematology analyzer (Drew Scientific, Inc., Oxford, CT) in accordance with manufacturer's recommendation.

### 2.5. Statistics

Data were presented as the mean and standard deviation. *P* values less than 0.05 in two-tailed student's* t*-test or one-way ANOVA were considered statistically significant.

## 3. Results

### 3.1. SJDBT Improves Cancer-Induced Weight Loss and Anorexia in CT-26 Tumor-Bearing Mice

**Figure 2:**
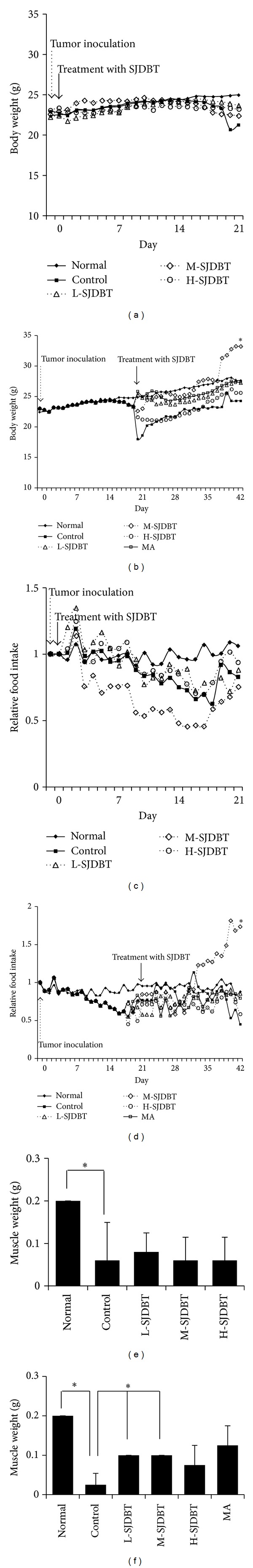
Effect of SJDBT on body weight and food intake in CT-26 tumor-bearing mice. ((a), (b)) Body weight of mice was measured every day at the same time. ((c), (d)) Food intake was measured every day at the same time. The measured quantity of food intake was divided by the number of total mice to determine each intake per animal a day. ◆: healthy normal mice (*n* = 5), ■: untreated control tumor-bearing mice (*n* = 5), △: low concentration (6.784 mg/kg) of SJDBT-treated tumor-bearing mice (*n* = 5), *⋄*: middle concentration (67.84 mg/kg) of SJDBT-treated tumor-bearing mice (*n* = 5), ○: high concentration (678.4 mg/kg) of SJDBT-treated tumor-bearing mice (*n* = 5), and □: megestrol acetate-treated tumor-bearing mice (*n* = 5). *P < 0.05.

To examine whether SJDBT prevents cancer-induced cachexia or treats cancer-induced cachexia, we divided into two groups (Figure 1). A day when CT-26 tumor cells were injected into mice was designated as day 0. We found that control groups in each experimental set compared to the normal group showed reductions of body weights by approximately 14.8% and 36.8%, respectively, at 21 days after tumor cell inoculations (Figures 2(a) and 2(b)), confirming that the injection of CT-26 tumor cells resulted in the loss of body weight. SJDBT was treated for 21 days prior to cancer-induced anorexia and cachexia in the prevention model and treated for 21 days after cancer-induced anorexia and cachexia in a therapy model. As shown in Figure 2(a), we found that L-SJDBT and H-SJDBT compared to the control increased body weights slightly by approximately 11.1% and 8.9%, respectively, while being not statistically significant. Otherwise, when SJDBT were administered into mice after cancer-induced weight loss, M-SJDBT-treated mice compared to the control significantly increased the weight by approximately 36.6% (Figure 2(b)). Therefore, SJDBT appeared to treat tumor-induced loss of body weight. Next, we performed experiments to determine whether SJDBT improves the appetite in a model of either prevention or treatment. L-SJDBT and H-SJDBT, compared to the control, ameliorated cancer-induced anorexia by approximately 25.3% and 25.1%, respectively, in the prevention model (Figure 2(c)). In addition, M-SJDBT compared to the control significantly increased food intake in the therapy model (Figure 2(d)). The control group compared to the normal decreased muscle weight by approximately 70% at 22 days after tumor cell injection and by approximately 87.5% at 43 days after tumor cell inoculation (Figures 2(e) and 2(f)). Both L-SJDBT and M-SJDBT prevented tumor-induced loss of muscle weight in the therapy model, since muscle weights of tumor-bearing mice treated with each of them were higher than those of tumor-bearing mice untreated by approximately 4-fold (Figure 2(f)). However, those were not effective in the prevention model (Figure 2(e)). Those results indicate that SJDBT may be effective for treating anorexia and for attenuating cachectic phenomenon, loss of muscle weight. Furthermore, our data showed that M-SJDBT was much more effective than MA in the treatment of cancer-induced reduction of food intake, while MA has been used to treat anorexia.

### 3.2. SJDBT Suppressed IL-6 and MCP-1 but Not TNF-*α* in the Therapy Model

**Figure 3:**
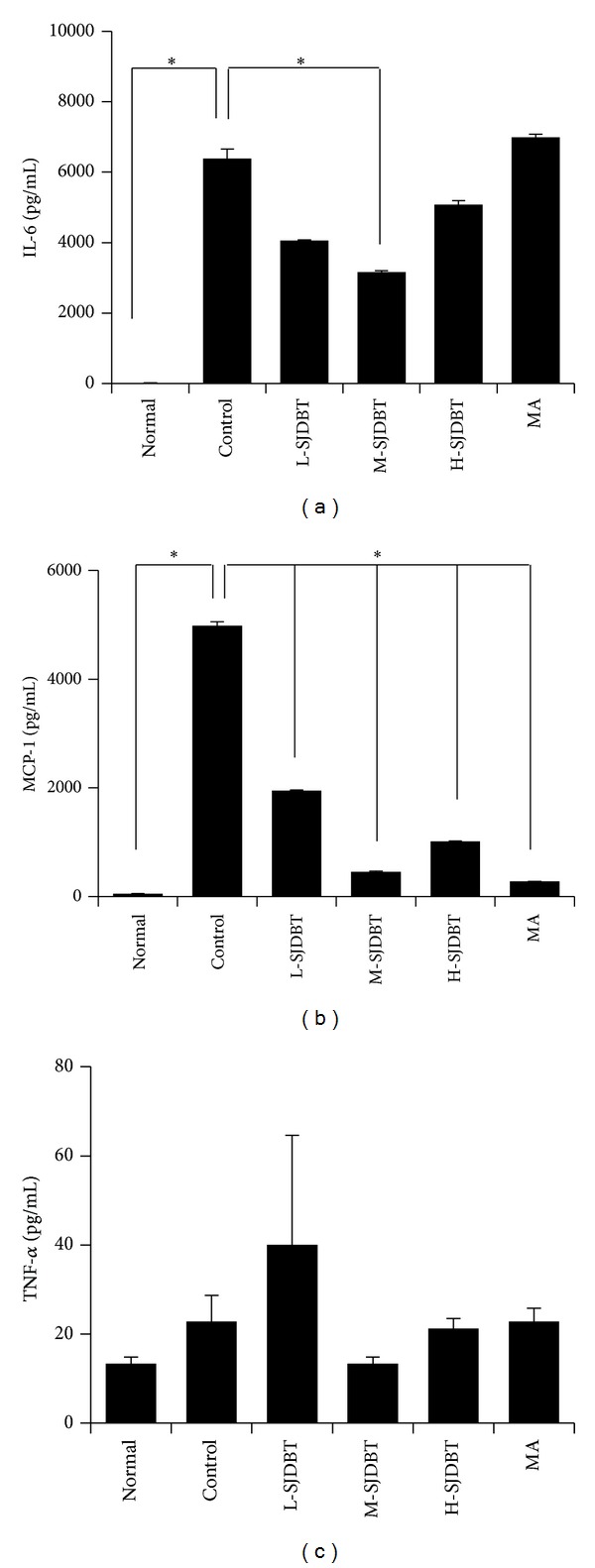
Effect of SJDBT on serums IL-6, MCP-1, and TNF-*α* levels in CT-26 tumor-bearing mice. ((a), (b)) 21 days after tumor cell injection, SJDBT were* p.o.* administrated every day until 42 days. At the end of experiments, mice were sacrificed. Blood samples were collected by cardiac puncture and serum samples were obtained blood sample after centrifuged. IL-6, MCP-1, and TNF-*α* level were measured using a Milliplex Mouse Metabolic Magnetic Bead Panel MMHMAG-44K-14. Experiments were performed in duplicate. Bars indicate means and standard deviations. *P < 0.05.

As proinflammatory cytokines such as interleukin-6 (IL-6), monocyte chemoattractant protein-1 (MCP-1), and tumor necrosis factor-*α* (TNF-*α*) derived from tumor cells were associated with cancer-related anorexia and cachexia [21, 22], we next examined whether SJDBT affects serum levels of IL-6, MCP-1, and TNF-*α*. Levels of IL-6 and MCP-1 significantly increased in the control compared to the normal by approximately 480-fold and 91-fold, respectively (Figures 3(a) and 3(b)). In addition, M-SJDBT significantly inhibited cancer-induced induction of IL-6 and MCP-1 by approximately 50.5% and 90.8%, respectively (Figures 3(a) and 3(b)). Furthermore, MCP-1 level was uniquely altered in all tested doses of SJDBT. Whereas the serum level of TNF-*α* increased slightly in the control compared to the normal by approximately 1.7-fold, M-SJDBT compared to the control reduced TNF-*α* level by approximately 41.4% while it was not statistically significant (Figure 3(c)). These results suggest that M-SJDBT may ameliorate cancer-induced anorexia and cachexia by altering levels of cytokines such as IL-6 and MCP-1.

### 3.3. Effects of SJDBT on Levels of Gut Hormones and Leptin in the Therapy Model

**Figure 4:**
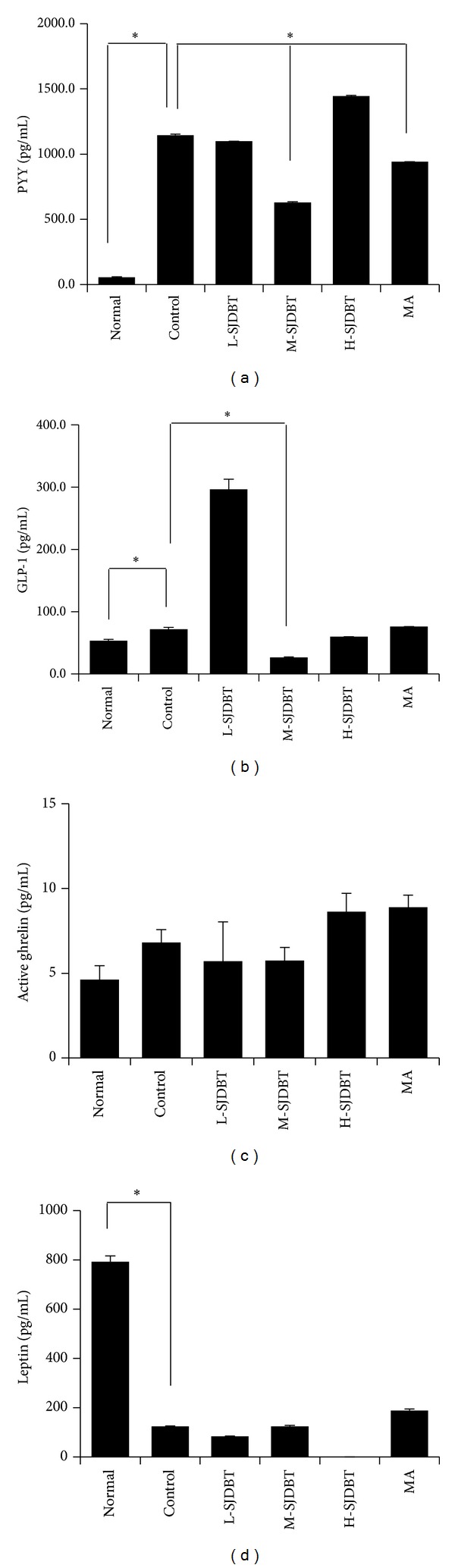
Effect of SJDBT on serums GLP-1, PYY, ghrelin, and leptin level in CT-26 tumor-bearing mice. ((a), (b)) 21 days after tumor cell injection, SJDBT were* p.o.* administrated for 21 days. At the end of experiments, mice were sacrificed. Blood samples were collected by cardiac puncture and serum samples were obtained blood sample after centrifuged. GLP-1, PYY, ghrelin, and leptin level were measured using a Milliplex Mouse Metabolic Magnetic Bead Panel MMHMAG-44K-14. Experiments were performed in duplicate. Bars indicate means and standard deviations. *P < 0.05.

As anorexia involves appetite-regulating hormones such as gut hormones (GLP-1, PYY, and ghrelin) and leptin [23, 24], we further examined whether SJDBT affects their levels in serum. Levels of GLP-1 and PYY significantly increased in the control compared to normal by approximately 1.3-fold and 20-fold, respectively (Figures 4(a) and 4(b)). M-SJDBT significantly inhibited cancer-induced induction of both GLP-1 and PYY by approximately 63.2% and 44.9%, respectively (Figures 4(a) and 4(b)). However, levels of ghrelin and leptin were not altered by M-SJDBT. Those results suggest that M-SJDBT regulation of GLP-1 and PYY may affect cancer-associated anorexia and cachexia.

### 3.4. SJDBT Improves Cancer-Induced Anemia in CT-26 Tumor-Bearing Mice

**Figure 5:**
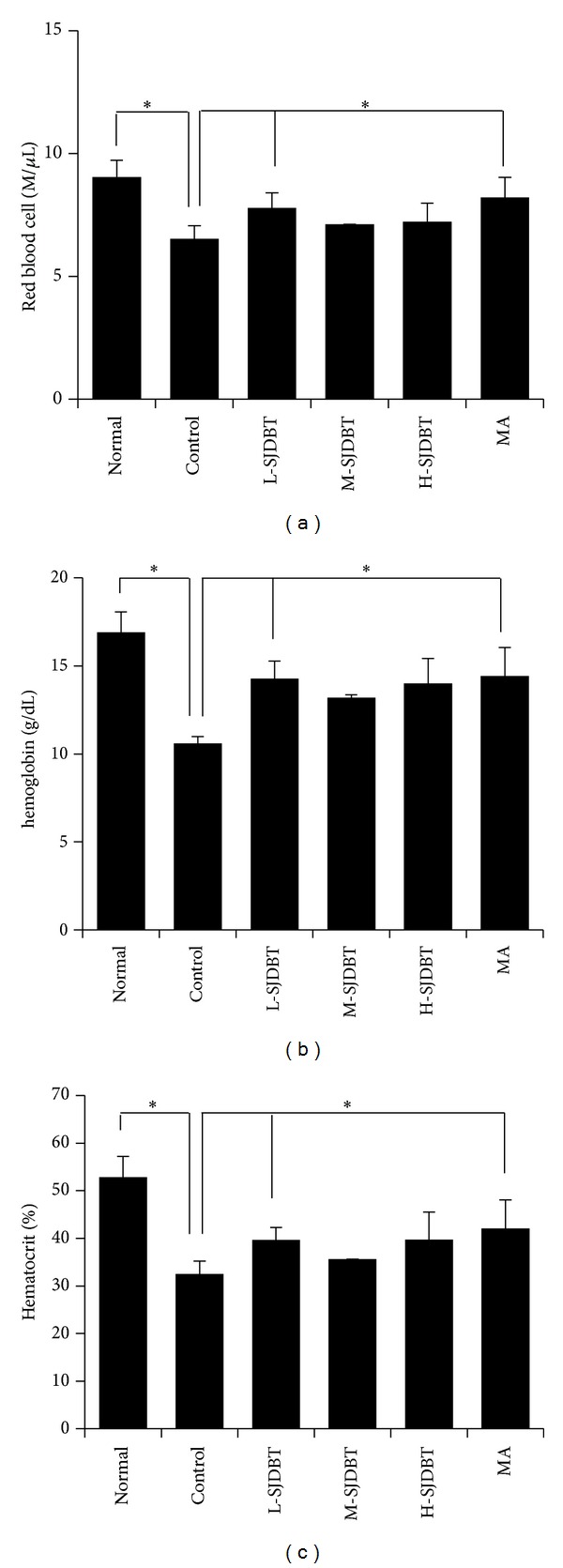
Blood analysis. ((a), (b), and (c)) 21 days after tumor cell injection, SJDBT were* p.o.* administrated for 21 days. At the end of experiments, mice were sacrificed. Whole blood samples were collected by cardiac puncture. The blood was placed in Vacutainer TM tubes containing EDTA (BD science, NJ, USA). Blood analysis was performed using the HEMAVET 950 hematology analyzer (Drew Scientific, Inc., Oxford, CT, USA) in accordance with manufacturer's recommendation.

**Figure 6:**
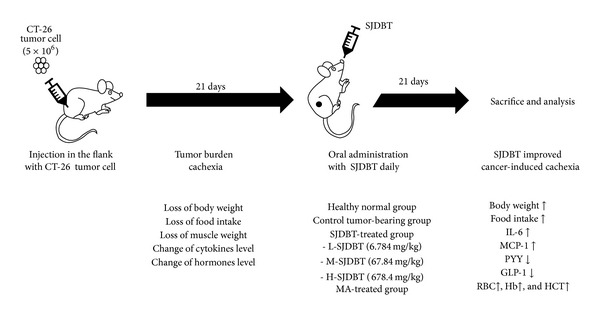
Schematic illustration. To set up the tumor burden cachexia mice model, mice were injected* s.c.* with CT-26 tumor cells. 21 days after tumor cell injection, three doses of SJDBT (L-SJDBT, M-SJDBT, and H-SJDBT) and MA were* p.o.* added daily. After treatment with indications for 21 days, mice were sacrificed and blood samples were analyzed for blood analysis and cytokines and hormones level in serum. SJDBT improved cancer cachexia in mice and inhibited the production of IL-6, MCP-1, PYY, and GLP-1. Therefore, we conclude that SJDBT improves cancer-induced cachexia including weight loss, anorexia, muscle wasting, anemia, and dysregulation of cytokines and hormones.

Because cancer-induced anorexia and cachexia are a multifactorial syndrome including anemia [25], we measured levels of red blood cells in blood samples using HEMAVET 950 hematology analyzer. The control compared to the normal accrued anemia, which was measured by numbers of red blood cells (RBC; normal mice versus control mice; 9.04 ± 0.68 M/*μ*L versus 6.53 ± 0.44 M/*μ*L; *P* = 0.02), hemoglobins (Hb; normal mice versus control mice; 16.91 ± 1.15 g/dL versus 10.6 ± 0.38 M/*μ*L; *P* = 0.0001), and hematocrits (HCT; percentage of red blood cells in whole blood; normal mice versus control mice; 52.9 ± 4.32% versus 32.53 ± 2.68%; *P* = 0.03). L-SJDBT compared to the control significantly increased values of RBC, Hb, and HCT by approximately 18%, 34.7%, and 22%, respectively (Figures 5(a), 5(b), and 5(c)). In addition, MA similarly affected those values, indicating that the treatment of anorexia might be linked to alterations of those values. While M-SJDBT compared to the control increased values of RBC, Hb, and HCT by approximately 7.6%, 24.5%, and 9.4%, respectively, those were not statistically significant. Therefore, those results indicate that L-SJDBT may be a good remedy for cancer-mediated anemia.

## 4. Discussion

As herbal medicine therapy has been known to be beneficial with high efficacy and safety, market of herbal medicine therapy is growing in Korea, China, and Japan as well as in the United States [26]. Especially, herbal medicines are used for clinical trial and therapy in cancer patients [27, 28]. However, biomedical scientists have limitations to use the herbal medicines due to the lack of evidence even in lab-based experimental studies [28]. For example, SJDBT is widely used for cancer patients suffering from symptoms such as loss of appetite, fatigue, and anemia in Korea, China, and Japan [10, 12, 13, 29]. However, its effect on the loss of either weight or food intake is still unknown. In this present study, we showed SJDBT effect on cancer anorexia and cachexia.

Cancer-induced anorexia and cachexia involve proinflammatory cytokines such as IL-6, MCP-1, and TNF-*α* [21, 22]. Furthermore, it has been suggested that MCP-1 rather than TNF-*α* is more important for cancer cachexia [22, 30, 31]. Circulating level of IL-6 is known to be important for keeping body weight and for survival in cancer patients [32–34]. In our study, SJDBT reduced IL-6 but increased MCP-1, while it did not affect TNF-*α*. Thus, it is plausible that SJDBT may modulate effects of cytokines on cancer-induced anorexia and cachexia. Meanwhile, GLP-1, PYY, ghrelin, and leptin are known as readouts for anorexia/cachexia [35]. SJDBT significantly suppressed GLP-1 and PYY levels, whereas it did not affect ghrelin and leptin. In sum, our data suggest that SJDBT ameliorates cancer-associated anorexia/cachexia by regulating cytokines (IL-6 and MCP-1) and hormones (GLP-1 and PYY). Anemia is tone of common features of cancer cachexia [6, 8, 36]. Especially, anemia is linked to the fatigue and QOL in cancer patients [36]. In addition, Hb level is supposed to be related to QOL [37]. SJDBT increased values of RBC, Hb, and HCT, which further indicates that SJDBT is beneficial for treating cancer anorexia/cachexia.

Cancer-associated anorexia is related to central mechanisms governing food intake [38]. Food intake is controlled by neuroendocrine paths through central nervous system (CNS) [39]. Short-term reaction for food intake is mediated by peptides produced by enteroendocrine cells in the gastrointestinal tract [39]. Incretin hormones such as GLP-1 mediate a response of the pancreas to nutrients, resulting in the reduction of food intake. Likewise, ghrelin is secreted from the stomach and promotes appetite. Leptin mediates long-term reaction for food intake. It is produced mainly from the adipose and stimulates food intake through CNS [40]. Furthermore, cancer-associated anorexia is caused by chronic stimulations via cytokines released from cancer cells or from host cells responding to cancer cells [41]. Cytokines have been known to interrupt with appetite-stimulating signaling [42]. Furthermore, cytokines impair muscle and fat metabolisms, which is tightly linked to cancer-associated anorexia [42, 43]. While cytokines such as TNF-*α* and IL-6 have been known to mediate cancer cachexia [42], SJDBT reduced IL-6 level without altering TNF-*α*. IL-6 is known to cause the loss of lean body weight in CT-26 tumor-bearing cachectic mice by inducing proteolytic pathways [44]. In addition, MCP-1, the circulating level of which was altered by SJDBT, has been known to regulate leptin level in the adipose and to mediate cachexia [22, 45]. Therefore, it is plausible that SJDBT may target cytokine production in cancer cells, which may result in modulations of hormone levels. However, we could not exclude possibilities that SJDBT may target multiple sources including cancer cells, cancer-associated immune cells, adipocytes, and hypothalamic neurons. Recent studies have revealed functional relationships between cytokines (IL-6 and MCP-1) and hormones (GLP-1 and PYY) [46–51]. Thus, it remains to be deciphered molecular and cellular mechanisms of SJDBT to ameliorate cancer-associated anorexia and cachexia.

## 5. Conclusion

Our study demonstrates that the SJDBT ameliorates cancer-induced anorexia and cachexia in CT-26 tumor-bearing mouse model by altering the production of IL-6, MCP-1, PYY, and GLP-1. Our* in vivo* studies first provide evidence on the role of SJDBT in cancer-associated anorexia/cachexia, which suggests that SJDBT may be useful for patients with cancer-associated anorexia/cachexia.
